# Extract of *Pleurotus pulmonarius* Suppresses Liver Cancer Development and Progression through Inhibition of VEGF-Induced PI3K/AKT Signaling Pathway

**DOI:** 10.1371/journal.pone.0034406

**Published:** 2012-03-28

**Authors:** Wenwen Xu, Jim Jun-hui Huang, Peter Chi Keung Cheung

**Affiliations:** 1 School of Life Sciences, The Chinese University of Hong Kong, Shatin, Hong Kong, China; 2 Marine Biology Institute, Shatou University, Shatou, Guangdong Province, China; Bauer Research Foundation, United States of America

## Abstract

Liver cancer or hepatocellular carcinoma is one of the leading causes of cancer-related deaths. Conventional chemotherapies are limited by the development of drug resistance and various side effects. Because of its non-toxicity and potent biopharmacological activity, metabolites derived from mushrooms have received more attention in cancer therapy. Our previous studies have demonstrated the anticancer effects of polysaccharide-protein complexes derived from the *Pleurotus* mushrooms. The aim of this study was to investigate the underlying molecular mechanism of the anticancer activity of a hot water extract containing a polysaccharide-protein complex isolated from *Pleurotus pulmonarius* (PP) in liver cancer cells. Our results indicated that exposure of liver cancer cells to PP not only significantly reduced the *in vitro* cancer cell proliferation and invasion but also enhanced the drug-sensitivity to the chemotherapeutic drug Cisplatin. Both oral administration and intraperitoneal injection of PP significantly inhibited the tumor growth in xenograft BALB/c nude mice. PP triggered a marked suppression of the PI3K/AKT signaling pathway in liver cancer cells *in vitro* and *in vivo*, and overexpression of the constitutively active form of AKT, Myr-AKT, abrogated this effect and the inhibited proliferation and invasion by PP. Both western blot and ELISA results showed that PP-treated liver cancer cells had reduced expression and secretion of vascular endothelial growth factor (VEGF). Addition of recombinant human VEGF attenuated the inhibitory effects of PP on PI3K/AKT pathway and the cancer phenotypes. Our results demonstrated that PP suppressed the proliferation, invasion, and drug-resistance of liver cancer cells *in vitro* and *in vivo*, mediated by the inhibition of autocrine VEGF-induced PI3K/AKT signaling pathway. This study suggests the potential therapeutic implication of PP in the treatment of human liver cancer.

## Introduction

Liver cancer is the fifth most prevalent cancer and the third leading cause of all cancer-related deaths in the world [1]–[3]. Unfortunately, the overall response rate of liver cancer to treatment is unsatisfactory mainly due to the late diagnosis and poor efficacy of therapies, especially the resistance to chemotherapeutic drugs and metastasis to other organs [4]. The alternations of multiple cell signaling pathways were frequently observed in a broad variety of human cancers, including liver cancer, in which the PTEN/PI3K/AKT signaling cascade has been reported to play a crucial role in the regulation of the malignant behaviors including proliferation, survival and invasion [5], [6]. Emerging evidences demonstrated that PI3K/AKT pathway can be regulated in an autocrine manner by various growth factors, such as insulin-like growth factor (IGF) and vascular endothelial growth factor (VEGF) [7], [8].

Natural phytochemicals and metabolites from plants are receiving increasing attention for their pharmacological effects in treatment and prevention of cancer because of their low toxicity and potential biological activity [9]. Both *in vitro* and *in vivo* studies have shown that these bioactive natural products can inhibit the initiation, promotion, and progression of carcinogenesis by interfering the signaling pathways in human cancer cells and their consumption has become a promising chemopreventive and chemotherapeutic strategy against cancers [10], [11]. Among them, edible mushrooms are known to be a rich source of anticancer agents, with their polysaccharides and polysaccharide-protein complexes being the most efficacious ones [12], [13]. Numerous reports have demonstrated the *in vitro* and *in vivo* anticancer activities of polysaccharides isolated from edible mushrooms such as *Lentinula edodes*, *Agaricus blazei* and *Phellinus linteus* against different human cancer cells [14]–[18]. A recent study demonstrated that crude extract of polysaccharide from *Phellinus linteus* inhibited the phosphorylation of AKT in breast cancer cells [19]. Crude extract of the polysaccharides present in *Ganoderma lucidum* was found to have anti-metastatic effects through the modulation of the PI3K/AKT pathway [20].

Recently, there are many *in vitro* studies demonstrating the antioxidant and anti-proliferative effect of crude extracts of polysaccharides and polysaccharide-protein complexes from the *Pleurotus* mushroom genus in different human cancer cells [2]–[25]. The antioxidant activity of many natural products has also been suggested to contribute to their anticancer effect [26]–[28]. Based on the antioxidant study of the mushroom species collection in our laboratory, an active polysaccharide and protein complex isolated from mushroom *Pleurotus pulmonarius*, coded as PP, was screened out for potential anticancer agent due to its significant antioxidant effect (Unpublished data) in this study. These intriguing preliminary findings have led us to investigate the underlying molecular mechanisms of the anti-carcinogenic effect by which PP exerted on two human liver cancer cells, Huh7 and Hep3B. These results demonstrated for the first time that inhibition of VEGF/PI3K/AKT cascade mediated the *in vitro* and *in vivo* suppressive effects of PP on the development and progression of human liver cancer cells. The chemosensitization of the liver cancer cells towards therapeutic drug cisplatin was also enhanced by PP treatment. In the present study, the therapeutic potential of PP in the treatment of human hepatocellular carcinoma was implicated.

## Results

### *In vitro* anti-proliferative effect of PP on liver cancer cells

**Figure 1:**
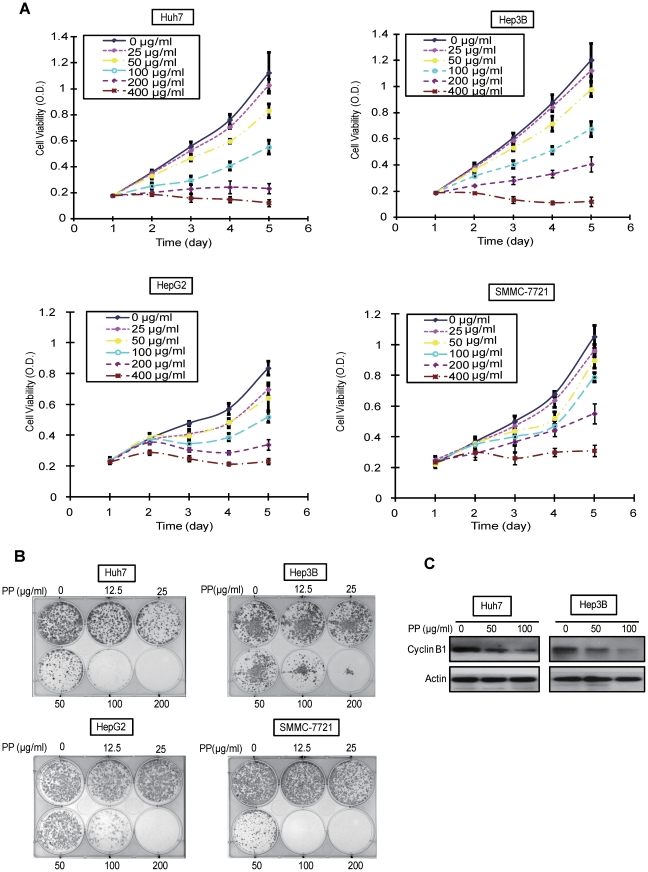
Effects of PP on the proliferation of liver cancer cell lines. **A.** MTT assay was taken to evaluate the liver cancer cell (Huh7, Hep3B, HepG2 and SMMC-7721) viability after the treatment with different concentrations of PP (0, 25, 50, 100, 200 or 400 µg/ml respectively) for up to 96 hr. Data are means (bars, SD) of three independent experiments. **B.** The colony-formation assay demonstrated a dose-dependent decrease in liver cancer cell proliferation (Huh7, Hep3B, HepG2 and SMMC-7721) when exposure to PP. **C.** Western blot was conducted and a dose-dependent reduction of cyclin B1 expression was observed. Actin was involved as a loading control.

To determine the inhibitory effect of PP on liver cancer cells, we first evaluated the growth and viability of four liver cancer cell lines (Huh7, Hep3B, HepG2 and SMMC-7721) with treatment of PP by using MTT assay. The results showed that PP exerted a significant inhibitory effect on Huh7, Hep3B, HepG2 and SMMC-7721 cells in dose- and time-dependent manner (Fig. 1A). This finding was further confirmed by colony-formation assay (Fig. 1B). Flow cytometry analysis showed that the treatment of liver cancer cells PP caused an accumulation of cells in the G2 phases (an increase from 12.42±0.25 to 19.66±1.12 in Huh7 and from 13.30±1.04 to 20.38±1.11 in Hep3B) (Table 1). In addition, consistent with the slightly increased sub-G1 population in PP-treated cells (Table 1), the important apoptosis markers, cleaved caspase3 and cleaved Poly (ADP-ribose) polymerase (PARP) were up-regulated in PP-treated cells, suggesting the proapoptotic effect of PP on liver cancer cells may be caspase-mediated. Western blot indicated a dose-dependent reduction in the expression of cyclin B1, an important G2 checkpoint protein, in the two liver cancer cells, suggesting that PP-induced G2 phase cell cycle arrest might be mediated by the down-regulated expression of cyclin B1 (Fig. 1C). Meanwhile, to exclude the possibility that the anti-cancer effect of polysaccharide-protein complex isolated from *Pleurotus pulmonarius* are non-specific, polysaccharide-protein complex isolated from another Pleurotus *mushroom, Pleurotus tuber-regium* (PTR) was applied as control, thus suggesting the specific effect of PP (Fig. S1).

**Table 1:** Representative cell cycle distribution by flow cytometry.

	Huh7			Hep3B		
PP (µg/ml)	0	50	200	0	50	200
SubG1 (%)	1.46±0.32	1.51±0.17	4.23±1.04*	1.08±0.34	1.76±0.55	3.36±0.60*
G0/G1 (%)	55.94±2.25	52.13±1.79	36.34±2.30**	62.64±1.95	56.16±1.11	36.23±1.25**
S (%)	30.16±2.01	30.87±2.09	39.75±2.24*	22.96±0.65	26.80±1.76	40.02±2.34**
G2 (%)	12.42±0.25	15.48±0.96	19.66±1.12**	13.30±1.04	15.27±1.98	20.38±1.11**

Huh7 and Hep3B cells were treated with PP (0, 50 or 100 µg/ml) for 48 hr, harvested and subjected to flow cytometry analysis of their DNA content. Data are means±SD of three independent experiments. Significant differences between the PP-treated and untreated cells were statistically analyzed by ANOVA (Tukey's multiple comparison test; *, *P*<0.05; **, *P*<0.005).

On the other hand, PP was examined for cytotoxic effects against normal liver cell WRL-68 by using MTT assay and colony formation assay. The results demonstrated that normal liver cell WRL-68 are more resistant to PP than liver cancer cells, suggesting the selective cytotoxicity of PP to cancer cells and its potential to be developed as anticancer agents (Fig. S2). To further investigate the potential implication of PP in the treatment of human cancers, we extended our studies in other cancer types. The results indicated a significant reduced proliferation rate in a dose-dependent manner upon PP treatment in human cancer cell lines from breast (T47D), lung (A549), stomach (AGS), and prostate (DU145) (Fig. S3).

### Effect of PP on the inhibition of VEGF expression and inactivation of PI3K/AKT pathway

**Figure 2:**
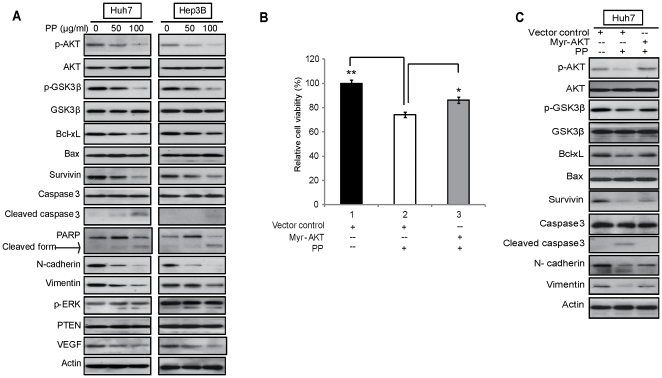
PP exerts its anticancer effect through inactivation of PI3K/AKT signaling pathway. **A.** Huh7 and Hep3B cells were treated with different concentrations of PP (0, 50 or 100 µg/ml) and cell lysis harvested at 48 hr for western blot analysis. PP treatment exerted a significant reduction in expression levels of p-AKT and its downstream targets including p-GSK-3β, Bcl-xL, and survivin. However, PP did not affect expressions of p-ERK and PTEN but VEGF expression was decreased in PP-treated cells. The experiments were repeated 3 times. **B.** Huh7 cells were transfected with AKT-overexpressing vector or the vector control, then treated with PP (50 µg/ml) and the cell viability measured by MTT at 72 hr. The results revealed an abrogated inhibitory effect in AKT-transfected cells when compared with the control. Data are given as percentage of untreated cells as control which was set at 100%. Data are means ± SD of three independent experiments. **C.** Huh7 cells were transfected with AKT-overexpressing vector or the vector control and then treated with PP. Cell lysate was harvested and applied to western blot. Significant differences between the PP-treated and untreated cells were statistically analyzed by ANOVA (Tukey's multiple comparison test; *, *P*<0.01; **, *P*<0.001).

Western blot showed that treatment of PP notably reduced protein expressions of phospho-AKT (p-AKT), the activated form of AKT, and its downstream targets including phospho-GSK3β (p-GSK3β), Survivin, Bcl-xL in a dose-dependent manner, but not that of Bax and total level of AKT and GSK3β in both liver cancer cells (Fig. 2A). In addition, consistent with the slightly increased sub-G1 population in PP-treated cells (Table 1), the important apoptosis markers, cleaved caspase3 and cleaved Poly (ADP-ribose) polymerase (PARP) were up-regulated in PP-treated cells, suggesting the proapoptotic effect of PP on liver cancer cells may be caspase mediated. Meanwhile, N-cadherin and Vimentin, which may account for reversal of epithelial-mesenchymal transition (EMT), were down-regulated in PP-treated cells (Fig. 2A). In contrast, expression level of phospho-ERK (p-ERK), another important signaling pathway involved in cancer cell regulation, was not substantially altered upon PP treatment, suggesting the specificity in the regulation of PI3K/AKT pathway by PP (Fig. 2A). Huh7 cells transiently transfected with AKT-plasmid consistently revealed an abrogated inhibitory effect when exposure to PP as indicated by MTT assay (Fig. 2B) and western blot analysis (Fig. 2C). These findings further confirmed that PI3K/AKT signaling pathway might contribute to the anticancer effects of PP in liver cancer cells.

### Effect of PP on the inhibition of VEGF secretion and inactivation of PI3K/AKT pathway

**Figure 3:**
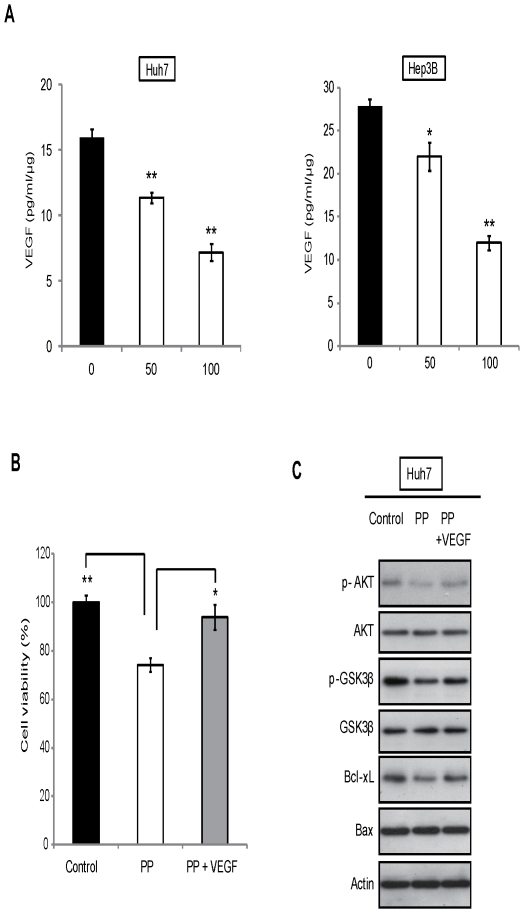
Inhibition of VEGF expression and secretion by PP mediates its effects on PI3K/AKT pathway. **A.** Effects of PP on secretion of VEGF in Huh7 and Hep3B cells. After the cells were treated by PP with various concentrations of (0, 50 or 100 µg/ml) for 48 hr, the conditioned medium were collected and VEGF level in the conditioned medium was determined by a human VEGF ELISA Kit. The result demonstrated a suppression of VEGF secretion in a dose-dependent manner upon PP treatment. **B.** MTT assay was performed to evaluate the proliferation when exposure to PP (50 µg/ml) in presence or absence of rhVEGF (0.6 ng/ml) for 72 hr in Huh7 cells. **C.** Huh7 cells were treated with PP (50 µg/ml) alone or the combination with rhVEGF (0.6 ng/ml), and cell lysate was harvested and applied to western blot. Data are expressed as mean ± SD. Significant differences between the PP-treated and untreated cells were statistically analyzed by ANOVA (Tukey's multiple comparison test; *, *P*<0.01; **, *P*<0.001).

It is interesting to find that PP reduced the expression of VEGF which is an important growth factor involved in proliferation and invasion of human cancer [29]. It has been well documented that PI3K/AKT pathway is activated in response to various growth factors [30]. Given that our western blot results showed reduced protein expressions of p-AKT and VEGF but not Phosphatase and tensin homolog (PTEN), an upstream protein of AKT (Fig. 2A), it is very likely that PP regulates PI3K/AKT pathway through a VEGF-mediated autocrine manner. Interestingly, ELISA data demonstrated a suppression of VEGF secretion in a dose-dependent manner upon PP treatment in Huh7 and Hep3B cells (Fig. 3A). Most importantly, addition of recombinant human VEGF (rhVEGF) at 0.6 ng/ml, the concentration reduced by PP compared to untreated group at 72 hr time point, attenuated the inhibitory effect of PP on cell proliferation (Fig. 3B) and PI3K/AKT signal pathway (Fig. 3C). These data suggest that the inactivation of PI3K/AKT pathway by PP may be mediated by the inhibition of VEGF in an autocrine manner.

### Effect of PP on enhancing the chemosensitivity of liver cancer cells to cisplatin

**Figure 4:**
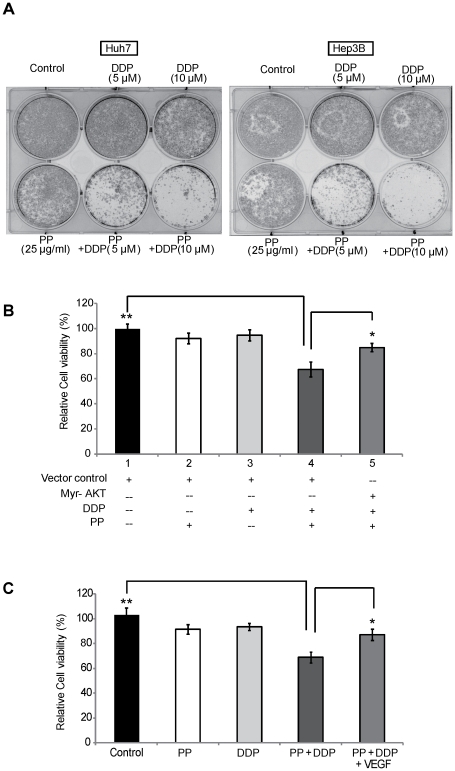
Synergistic effect of PP with cisplatin (DDP) in liver cancer cells. **A.** Huh7 and Hep3B cell were treated with DDP (up to 10 µM), PP (25 µg/ml), or the combination and then colony formation assay performed. **B.** Huh7 cells were transfected with AKT-overexpressing vector or the vector control, then treated with low dosage of PP (25 µg/ml), DPP (5 µM), or the combination for 72 hr and the cell viability measured by MTT assay. **C.** MTT assay was performed to evaluate the proliferation when exposure to low dosage of PP (25 µg/ml), DDP (5 µM) alone or the combination, in the absence or presence of rhVEGF for 72 hr in Huh7 cells. Addition of rhVEGF (0.6 ng/ml) led to an abrogated sensitization. Data are given as percentage of untreated controls which were set at 100%, with mean ± SD of at least three independent experiments. Significant differences between the PP-treated and untreated cells were statistically analyzed by ANOVA (Tukey's multiple comparison test; *, *P*<0.01; **, *P*<0.001).

A large body of evidence has indicated that PI3K/AKT pathway critically contributes to drug resistance in human cancer [31] and inactivation of PI3K/AKT by PP might lead to alternation of drug sensitivity in liver cancer cells. From the colony formation assay, cisplatin (DDP) at 5 µM or 10 µM only slightly decreased the cell viability, whereas a significant synergistic effect on colony formation in Huh7 and Hep3B cells was observed when DDP was combined with a low concentration of PP (25 µg/ml) (Fig. 4A), suggesting that PP might be a sensitizer for the chemotherapeutic drug. Transfection of AKT plasmid (Fig. 4B) and addition of rhVEGF (0.6 ng/ml) (Fig. 4C) resulted in an abrogated sensitization, further supporting the vital role of AKT pathway and VEGF played in the regulation of liver cancer cells by PP.

### PP decreases the invasive potential of liver cancer cells

**Figure 5:**
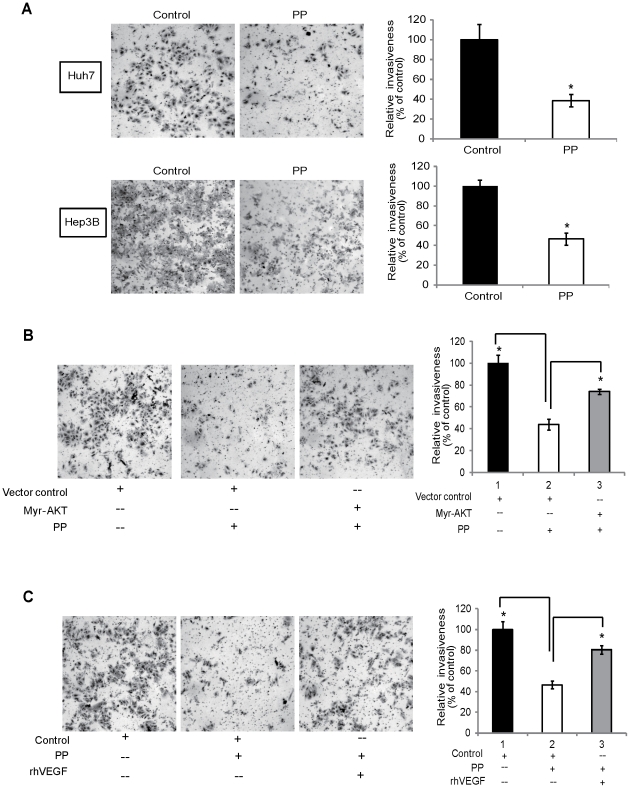
Effect of PP on invasive potential of liver cancer cells. **A.** Boyden chamber assay was used to determine the invasiveness of Huh7 and Hep3B cells upon treatment with control or PP (50 µg/ml) PP for 24 hr. PP significantly suppressed liver cancer cell invasion. Right panel showed the quantification by submerging the chambers in 1% SDS and measuring the absorbance of the solution at 570 nm. The mean of triplicate assays for each experiment condition was calculated and values normalized to the untreated cells. **B.** The Huh7 cells transfected with AKT-overexpressing vector, or the vector control were treated with PP, and the cell invasiveness was tested. Right panel shows the quantification. **C.** Huh7 cells were treated with PP in the absence or presence of 0.6 ng/ml rhVEGF, and the invasive potential determined. Right panel shows the quantification. Data are expressed as mean ± SD. Significant differences between the PP-treated and untreated cells were statistically analyzed by ANOVA (Tukey's multiple comparison test; *, *P*<0.01; **, *P*<0.001).

Using chamber invasion assay, it was found that PP at a low dosage (50 µg/ml) which had no significant effects on cell proliferation at 24 hr time point markedly decreased the invasiveness of Huh7 and Hep3B cells (Fig. 5A). Interestingly, the inhibitory effect of PP on the invasion of Huh7 was attenuated by overexpression of constitutively activated AKT (Fig. 5B) and addition of rhVEGF (Fig. 5C), further demonstrating the involvement of VEGF/PI3K/AKT cascade in the regulation of liver cancer cells by PP. In addition, a dose-dependent reduced expression levels of N-cadherin and Vimentin, the important makers for cell migration and invasion, was noted in western blot analysis shown in Fig. 2A, supporting the observed inhibitory effect of PP on the invasiveness of liver cancer cells.

### PP significantly suppresses human liver cancer xenograft growth in nude mice

**Figure 6:**
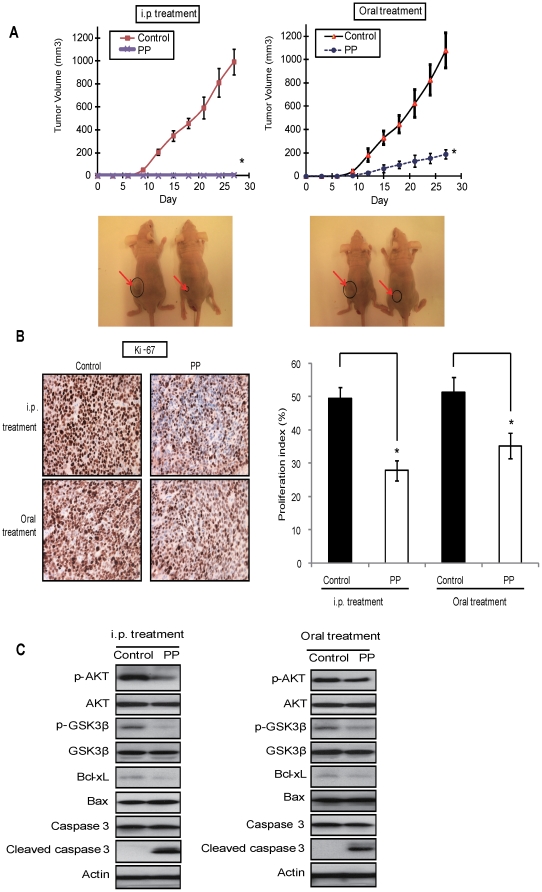
PP suppressed tumor xenograft growth of Huh7 cells in nude mice model. **A.** Growth curves and representative photographs of nude mice treated with i.p (left panel) or oral administration (right panel) of PP with the vehicle control (water). Point represents the mean tumor size with the equation V = (length×width^2^)/2. **B.** The tumors harvested from water-treated or PP-treated mice were fixed, embedded, sectioned, and applied to Ki-67 immunohistochemical staining and quantification. The number of positively stained nuclei in a minimum of six randomly selected fields from representative tumor sections divided by the total number of cells is presented in the right panel. **C.** The tumor tissues were harvested for western blot. Data are expressed as mean ± SD. Significant differences between the PP-treated and untreated cells were statistically analyzed by ANOVA (Tukey's multiple comparison test; *, *P*<0.01; **, *P*<0.001).

Considering the above *in vitro* results showing that PP reduced the proliferation of liver cancer cells, it will be crucial to evaluate whether PP could inhibit tumorigenicity of liver cancer cells *in vivo*. Firstly, nude mice with established s.c tumor xenograft from Huh7 cells was employed and treated with either oral administration or intraperitoneal (i.p.) injection of PP. Both the oral feeding and i.p injection of PP significantly (p<0.001) impaired the tumor growth (Fig. 6A) although the effect by oral administration (inhibition ratio ∼82%) was less pronounced comparing with i.p injection (inhibition ratio ∼99%).

Tumor xenograftes harvested from the four groups of mice were subjected to immunohistochemistry and western blot analysis. A significantly lower percentage of Ki-67-positive cells found in PP-treated group compared with control group (Fig. 6B) suggested that the inhibitory effect of PP might be due to decreased cell proliferation. The alternation of protein expression in the PP-treated xenograft animals, including a down-regulated expression of p-AKT, P-GSK3β, Bcl-xL, and an up-regulated expression of cleaved caspase-3 in these *in vivo* experiments (Fig. 6C) were consistent with those *in vitro* studies (Fig. 2A).

**Figure 7:**
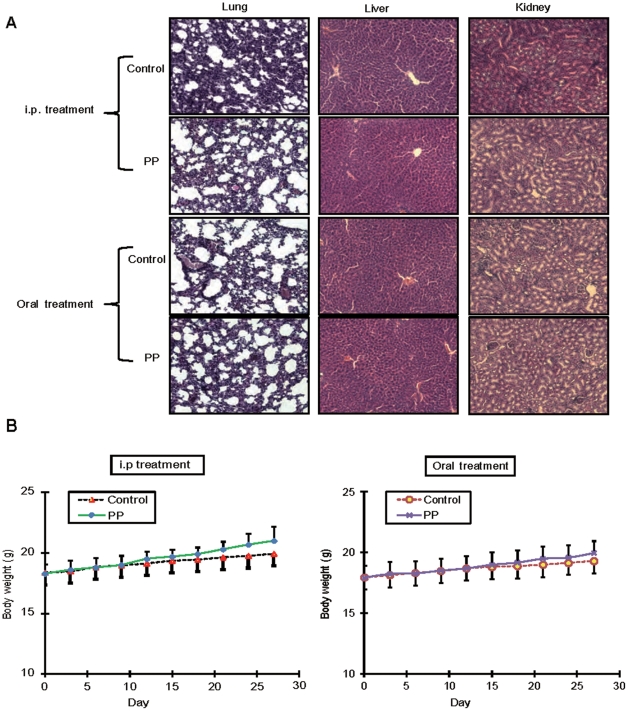
No obvious adverse effects find in PP-treated mice. **A.** Hematoxylin and eosin (H&E) staining of lung, liver, kidney from the four indicated groups of mice. **B.** Body weight (gram) of the mice treated with PP and control.

Furthermore, no obvious side effects or changes were observed by comparing the histological features of internal organs including the liver, lung and kidney (Fig. 7A) and body weight of the animals (Fig. 7B) in the treatment and control groups, suggesting the safety and clinical implication of PP as an anticancer agent.

## Discussion

In this study we demonstrated that PP, a hot water extract containing a polysaccharide-protein complex isolated from *Pleurotus pulmonarius* inhibited the development and progression of liver cancer cells *in vitro* and *in vivo*. Such anticancer effects are through the inhibition of VEGF-mediated autocrine regulation of PI3K/AKT pathway. The significance of these findings is discussed below.

Firstly, we demonstrated that treatment of PP reduced tumorigenicity of liver cancer cells in the xenograft nude mouse model without any obvious side effect. Despite the advancement in chemotherapeutics in recent years, chemotherapy is still associated with serious side effects, such as nephrotoxicity, nausea, hair loss, skin irritation, anemia, infertility [32], [33]. Therefore, naturally occurring anticancer compounds present in human diets have important implication of chemotherapy and chemoprevention, especially for those with low toxicity and high potency such as resveratrol [34] and tea polyphenols [35]. More interestingly, the *Pleurotus pulmonarius* fruit body extracts have been reported to delay the progression of chemically induced hepatocellular carcinoma in CBA mice [36]. In this study, we found that both i.p injection and oral administration of PP remarkably inhibited the tumor growth. The comparatively less pronounced tumor inhibition observed in oral administration of PP might be due to the pharmacokinetic limitations and intestinal degradation of the compounds [37]. No significant differences were found by comparing the histology of lung, liver, kidney and body weight in the PP-treated and control mice, suggesting that there was no observable adverse health effect on mice treated with PP. The remarkable anticancer effects and low toxicity to animals lend support to the feasibility of PP for human liver cancer treatment.

Secondly, the present results indicated that PP inhibited the proliferation rate of liver cancer cells, and this effect may be through G2 cell cycle arrest. Interestingly, besides the G2 phase, the simultaneous accumulations in S phase were observed, the possible molecular mechanisms warrant further investigation. In addition, our data showed that PP significantly increased the drug sensitivity of liver cancer cells to cisplatin (Fig. 4). Despite the recent advancement in the development of chemotherapies in the past decades, most patients eventually relapsed after treatment, implying that tumors acquired mechanisms of drug resistance, which crucially contributed to the high mortality of liver cancer [38]. In order to overcome chemoresistance, chemosensitization which is based on the combination of another drug by regulating one or more mechanisms of resistance has been taken as one attractive strategy, especially the involvement of natural existing compounds [39]. Interestingly, a large body of evidence is emerging to indicate that several signaling pathways are critically involved in mediating acquired resistance to chemotherapeutic drugs in human cancer, including PI3K/AKT, MEK/ERK, JNK and AMPK pathways [40], [41]. The inhibition of PI3K/AKT pathway by pharmacologic approach could lead to restoration of drug sensitivity [42], [43]. In this regard, inactivation of PI3K/AKT by PP (Fig. 2 and 3) suggested that exposure to PP might enhance the drug susceptibility of liver cancer cells. Furthermore, the fact that PP-induced down-regulation of Bcl-xL and Surviven (Fig. 2A), both of which are important makers of multi-drug resistance, further showed the promising role of PP as chemosensitizer.

Another highlight should be addressed is that the PP treatment resulted in a reduced invasiveness of liver cancer cells (Fig. 5A), which was further supported by the decreased protein expressions of N-cadherin and vimentin in PP-treated cells (Fig. 2A). It had been reported that N-cadherin is crucial for tumor-vessels interactions, extravasation and metastatic dissemination [44], [45] and vimentin is widely used as a marker accounting for the epithelial to mesenchymal transition (EMT) [46], [47]. EMT is a trans-differentiation program required for tissue morphogenesis during embryonic development. However, it can adversely cause organ fibrosis and promote carcinoma progression through a variety of mechanisms, acquiring invasive and metastatic properties [48]–[50]. The investigation of EMT inhibitor has opened new door for controlling metastasis which crucially contributes to the high mortality rate of liver cancer [51]. Therefore, the present findings in the inhibitory effects of PP on the invasive potential of liver cancer cells have clinical implication in the treatment of liver cancer.

Besides the functional study, elucidation of the mechanisms is crucial for understanding the PP as potential anticancer agent. Since the constitutive activation of PI3K/AKT signaling cascade is frequently observed in human cancer phenotypes and crucially related with cancer cell proliferation, survival, invasion and angiogenesis, the inhibition of PI3K/AKT pathway has become an attractive approach for liver cancer treatment [52], [53]. AKT inhibitors have been reported to suppress the tumor growth in liver, suggesting the rational for targeting the PI3K/AKT pathway in this disease. However, the employment of AKT inhibitor is associated with unexpected side effects [54]. *Cordyceps militaris*, a medicinal mushroom, was reported to induce cell cycle arrest and apoptosis through inactivation of PI3K/AKT pathway in breast cancer [55]. Also, oral administration of a polyphenolic fraction isolated from green tea significantly inhibits prostate cancer development and metastasis through PI3K/AKT pathway in transgenic adenocarcinoma of mouse model [56]. Interestingly, our result showed that PP down-regulated the expression of p-AKT, the active form of PI3K/AKT, in a dose dependent manner, in line with the result of functional study. In addition, the results of parallel experiments by transfection of AKT plasmid consistently illustrated an abrogated inhibitory effect of PP, further confirming the involvement of PI3K/Akt signaling cascade (Fig. 2B and 2C).The deregulation of PI3K/AKT pathway significantly contributed to the development of hepatocellular carcinoma [57]. Personalized approaches based on a refinement of the molecular classification of HCC, have been taken as potential strategies in the clinical treatment of HCC [58]. Meanwhile, the identification of reagents that interfere with signaling pathways which contribute to HCC might lead to the development of personalized therapies for the patients, and thereby maximize the efficacy and cost benefit [59]. This study provides a novel potential therapeutic option for the patients presented with constitutive PI3K/AKT activation. On the other hand, although no obvious alternations were observed in ERK pathway when exposure to PP, the effect of PP on other signaling pathways warrants further investigation.

Interestingly, it was found that VEGF was down-regulated at both protein expression and secretion levels upon PP treatment. In addition, stimulation of the cells with rhVEGF in the culture medium attenuated the inhibitory effect of PP on AKT pathway and cancer phenotypes (Fig. 3). Carcinogenesis is a multi-step process mediated by many growth factors including placental growth factor (PIGF), platelet-derived growth factor (PDGF), and Vascular Endothelial Growth Factor (VEGF) [60]. In the tumor microenvironment, the various growth factors control not only the cancer cells but also the stromal in autocrine and paracrine manner [61], [62]. VEGF is one of the key factors responsible for angiogenesis which controls the formation of new blood vessels from existing vasculature, and plays an essential role in tumor growth, invasion and metastasis [63], [64]. There are studies demonstrated that the extract of *Phellinus linteus* could significantly suppress the angiogenesis through the downregulation of VEGF secretion by targeting AKT in breast cancer or through Wnt/β-catenin signaling pathway in colon cancer [65], [66]. Anti-angiogenic therapy based on inhibition of VEGF/VEGF receptor is believed to be powerful clinical strategy in the treatment of cancer, especially for liver cancer which is a highly vascular tumor [67], [68].The inhibitory effect of PP on VEGF suggests the anti-angiogenic activities of PP and its potential implication in anti-angiogenic therapy. In this study, the new findings related to the inhibition of VEGF/PI3K/AKT cascade in liver cancer cells by PP treatment provide insights into the molecular mechanisms to support its potential clinical application.

In conclusion, PP, a *Pleurotus* mushroom polysaccharide-protein complex, has both *in vitro* and *in vivo* anticancer activities against liver cancer cells through the inhibition of secretory VEGF-mediated autocrine regulation of PI3K/AKT signaling. Further studies on the anticancer effect of the *Pleurotus* mushroom polysaccharide-protein complex on liver cancer stem cancers will be carried out to evaluate its potential application in cancer treatment and prevention.

## Materials and Methods

### Preparation and characterization of mushroom hot water extract

Edible mushroom (*Pleurotus pulmonarius*) was purchased from the local supermarket and the species was positively identified based on its morphology. Dried powder was prepared from grinding the freeze-dried mushroom fruiting bodies into particle size less than 0.5 mm and then subjected to a hot water extraction at 95–100°C for 3 hr with a sample-to-solvent ratio of 1∶25 (w/v). The mixture was centrifuged 10000 *g* at 4°C for 30 min and the supernatant was dialyzed with dialysis membrane tubing (molecular weight cut-off of 6000 to 8000, Spectrum Laboratories, CA) and freeze-dried to give the hot water extract designated as PP For cell culture study, a solution of PP was sterilized with 0.22 µm pore size filter prior to use. Limulus Amebocyte Lysate test kit (Sigma, MI) was used to show that endotoxin contamination was below 0.05 EU/ml in PP. Phenol-sulfuric acid method [69] and the Lowry method [70] were used to determine the contents of polysaccharide and protein, respectively. The composition of the monosaccharide in PP was measured by gas chromatography by procedures described previously [71]. The content of polysaccharide and protein in PP was found to be 85.07±2.20 and 11.85±0.35% of dry matter (n = 3) with a monosaccharide profile containing mannose, glucose and galactose with a ratio of 10∶5∶2. Meanwhile, the polysaccharide-protein complex from another *Pleurotus* mushroom, *Pleurotus tuber-regium*, was also isolated by using the same protocol, coded as PTR.

### Cell culture and chemotherapeutic drugs

Human liver cancer cell lines (Huh7, Hep3B, SMMC-7721 and HepG2) were maintained in DMEM (Sigma) supplemented with 10% fetal bovine serum (FBS) (Invitrogen, Carlsbad, CA) and 1% (w/v) penicillin-streptomycin (Invitrogen), and human breast cancer (T47D), lung adenocarcinoma (A549), human stomach adenocarcinoma (AGS), prostate cancer (DU145) cell lines and normal liver cell line (WRL-68) were cultured in RPMI-1640 (Sigma). All cancer cell lines were obtained from ATCC. The chemotherapeutic drug Cisplatin (DDP) was purchased from Calbiochem (Darmstadt, Germany). Recombinant human VEGF (rhVEGF) was purchased from Peprotech (Rocky Hill, NJ).

### Plasmid transfection

The plasmid expressing constitutively activated, myristylated form of AKT (Myr-AKT), was kindly provided by Dr L Larue (Developmental Genetics of Melanocytes, CNRS-Institut Curie). The plasmid or vector control was transiently transfected into Huh7 cells using Lipofectamine 2000 transfection reagent (Invitrogen) according to the manufacture's instruction. In brief, Lipofectamine 2000 reagent and the plasmids were diluted in Opti-MEM medium (Invitrogen) and then mixed together followed by 20 min of incubation at room temperature. Next, the mixtures were added into the cell culture flask and the culture medium was replaced 24 hr after transfection. The transfected cells were harvested at indicated time point for analysis.

### 3-(4, 5-Dimethyl thiazol-2-yl)-2,5-diphenyl tetrazolium bromide (MTT) assay

Cell viability was measured using MTT proliferation assay. Briefly, about 1000 cells were seeded in a 96-well plate for 24 hr before PP was added. Cell viability was determined at 0 hr, 24 hr, 48 hr, 72 hr and 96 hr after treatment of PP. Twenty microliters of MTT reagent (5 mg/ml in PBS; Sigma) was added at indicated time point and the cells were incubated for 4 hr at 37°C. Then, the formazan crystals were dissolved by adding 200 µl DMSO and taken for optical density (O.D.) measurement at a wavelength of 570 nm. Each data point represented the average of three independent experiments.

### Colony-formation assay

About 1000 cells were seeded in a 6-well plate for 24 hr before addition of PP or cisplatin. After about 2 weeks, the cells were fixed in 75% ethanol and stained with 0.1% (v/v) crystal violet. The experiments were repeated three times.

### *In vitro* cell invasion assay

BioCoat matrigel invasion chambers (BD Biosciences, Bedford, MA) were used to investigate the cell invasive potential. Briefly, the chambers were incubated with serum-free DMEM medium for 2 hr at 37°C for rehydration prior to use. About 5×10^4^ cells in 0.5 ml serum-free DMEM medium were delivered into the upper compartment while the lower compartment was filled with complete DMEM medium containing 10% FBS as a chemoattractant. After incubation for 24 hr at 37°C and removal of the cells remaining on the upper surface of the chamber with cotton swabs, the invaded cells adhering to the bottom surface of the chamber membrane were fixed in methanol and stained with 0.1% (v/v) crystal violet for 10 min. Images of three different fields were captured from each membrane. The quantification of the invaded cells was made by submerging the chambers in 1% SDS and measuring the absorbance of the solution at 570 nm. The mean of triplicate assays for each experimental condition was calculated and values normalized to the untreated cells.

### Cell cycle analysis

Cells (1×10^6^) were seeded in a 6-well culture plate for 24 hr and treated with of PP. After 48 hr, cell pellets were collected by trypsinization, fixed in 75% ethanol and kept at 4°C overnight. The cell pellets were resuspended with suitable propidium iodide (50 µg/ml) and RNase (1 µg/ml) solution, then incubated for 30 min at 37°C in a water bath. The cell cycle distribution was determined by the flow cytometry analysis system (Beckman Coulter, XL-MCL, Miami, FL) and analyzed by using Multicycle Analysis AV software (Pheoenix Flow Systems, SanDiego, CA).

### Western blot analysis

The control and treated cells were harvested at indicated time points and applied for gel electrophoresis. Briefly, the cell pellets were treated with lysis buffer (Cell Signaling Technology, Beverly, MA) with 1% phenylmethanesulfonyl fluoride (PMSF), and kept on ice for 30 min followed by centrifuge at 10000 *g* for 30 min at 4°C. The protein concentration in the supernatant collected was determined by BCA protein assay kit (Thermo, Rockford, IL). An appropriate amount of protein was mixed with protein loading buffer [16% (w/v) SDS, 48% (v/v) glycerol, 2.4% (w/v) Tris base, 8% (v/v) β-mercaptoethanol and 0.1% (w/v) bromophenol blue] and boiled for 10 min at 95°C, then loaded into SDS-polyacrylamide gel (4% stacking gel and 12% separating gel) for electrophoresis. The gel was transferred to PVDF membrane and the membrane was blocked with 5% fat-free dry milk in Tris-Buffered Saline Tween-20 (TBST) for 1 hr at room temperature before being washed with TBST and probed with primary antibodies. The following primary antibodies were used: Bcl-xL, Bax, caspase-3, cleaved caspase-3, p-AKT, AKT, p-GSK3β, GSK3β, Vimentin, PTEN, Survivin and poly(ADP-ribose) polymerase (PARP) (Cell Signaling Technology); N-cadherin, actin and VEGF (Santa Cruz Biotechnology, Santa Cruz, CA); and p-ERK (BD Biosciences). After washing, the membrane was incubated with respective horseradish peroxidase (HRP) conjugated secondary antibodies. The signals of immune blot were developed by ECL Plus Western blotting detection system (Amersham, Piscataway, NJ) and visualized by exposing a medical X-ray film (Fuji, Japan) onto the membrane.

### VEGF ELISA Kit

Human VEGF Enzyme-Linked Immunosorbent Assay kit (Pierce Biotechnology, Rockford, IL) was used to determine the human VEGF concentration in the cell-free culture medium. According to the manufacture's instruction, briefly, the conditioned medium treated by PP or control was collected at indicated time point and added into a microplate coated with anti-human VEGF_165_ antibody. After the unbound proteins were removed, a biotinylated detecting antibody was added and bound to VEGF. Excess detecting antibody was removed and streptavidin-horseradish peroxidase was added to react with TMB Substrate Solution. The color intensity of the sample was measured at 450 nm and level of VEGF was determined by the ratio of the absorbance between the treated and untreated cells.

### Tumor xenograft nude mouse model

All animal care and experimental procedures were approved by the Department of Health of the Hong Kong Special Administrative Region in China under the Animals (Control of Experiments) Ordinance Chapter 340 (Permit number DH/HA&P/8/2/1 Pt. 5). Twenty-four 6–8 weeks old male nude BALB/c mice were supplied by Animal House of The Chinese University of Hong Kong and randomized into 4 groups (n = 6 per group) and 5×10^6^ Huh7 cells in PBS mixed with equal volume of Matrigel (BD Biosciences) were subcutaneously injected into the flank of nude mice. Five days after the cancer cell inoculation, the mice were force-fed daily with an aqueous solution of PP (200 mg/kg) (group **1**) and water as control (group **2**). PP with a dose of 50 mg/kg was also intraperitoneally (i.p) injected into the tumor-bearing mice (group **3**) thrice a week, whereas the control (group **4**) received the vehicle only. The tumor volumes and body weight were measured every three days and tumor volumes were calculated with the equation V = (length×width^2^)/2. The experiments were carried out for four weeks but if the tumor size reached 1500 mm^3^ during this period of time, the experiment was terminated. All surgery was performed under sodium pentobarbital anesthesia, and all efforts were made to minimize suffering. At the end of the experiment, the tumors were harvested for western blot and immunohistochemical analysis. The vital organs (liver, lung, and kidney) were fixed in 4% paraformaldehyde, paraffin-embedded and stained by haematoxylin and eosin (H&E).

### Immunohistochemistry

Immunohistochemistry was performed as described previously [72]. In brief, the paraffin-embedded sections from tumor xenograft were deparaffinized followed by rehydration and incubation with 0.3% hydrogen peroxide for 30 min. Next, antigen retrieval was performed in a microwave oven. The slides were blocked with normal serum, followed by incubation with primary antibody against Ki-67 (Dako Mississauga, ON) at 4°C overnight. After the application of the biotinylated secondary antibodies, including peroxidase-conjugated avidin-biotin complex and 3, 3′-diaminobenzidine (Dako) as chromogen, counterstaining with the H&E was performed. To determine the ki-67 proliferation index, the number of positively stained nuclei in a minimum of six randomly selected fields from representative tumor sections by the total number of cells was counted.

### Statistical Analyses

The experiments were repeated at least three times. The data from each experiment were presented as the mean ± standard derivation (SD). The means among different groups were evaluated using ANOVA followed by Tukey's multiple comparison tests for significant differences with P values<0.05.

## Supporting Information

Figure S1 **No obvious inhibitory effect in liver cancer cell proliferation upon treatment with extract of another Pleurotus mushroom,** ***Pleurotus tuber-regium*** **.** MTT assay was applied with polysaccharide and protein complex from another mushroom *Pleurotus tuber-regium* (PTR) as control.
(PDF)

Figure S2 **Potential cytotoxic activity of PP in normal cells.** **A.** MTT assay was applied to determine the potential cytotoxic effect of PP against normal liver cell line WRL-68. **B.** Colony-formation assay demonstrated that WRL-68 cells are more resistant to PP than liver cancer cells.
(PDF)

Figure S3 **Effects of PP on the proliferation of other cancer cell lines.** **A.** Colony-formation assay was used to test the anti-proliferative effect of PP in other cancer types, including lung cancer cells (A549), stomach cancer cells (AGS), prostate cancer cells (DU145) and breast cancer cells (T47D).
(PDF)
