# Supplementary figures and images for: Extract of Pleurotus pulmonarius Suppresses Liver Cancer Development and Progression through Inhibition of VEGF-Induced PI3K/AKT Signaling Pathway
Source: PLoS One. 2012 Mar 28;7(3):e34406. doi: 10.1371/journal.pone.0034406 (PMC3314644; doi:10.1371/journal.pone.0034406)

A

Huh7

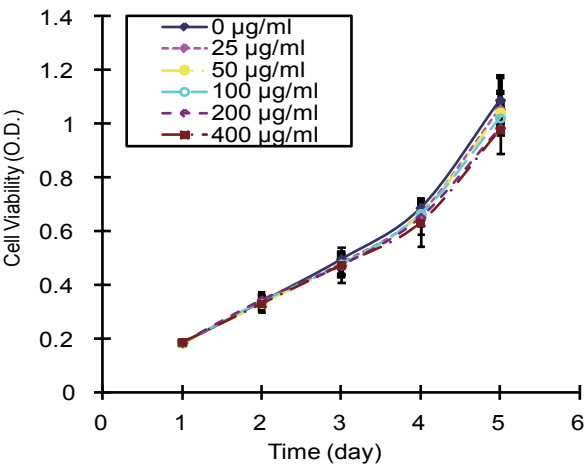

Hep3B

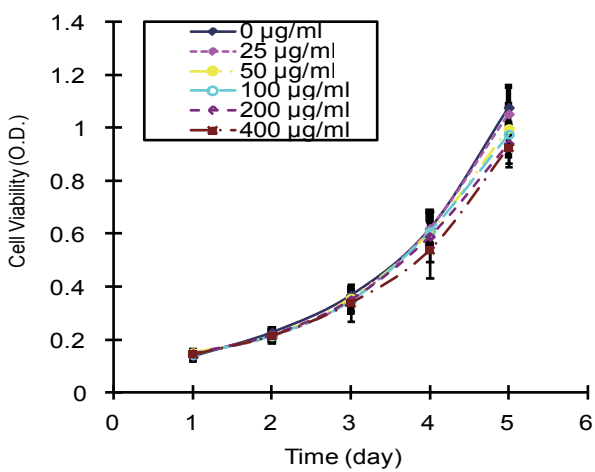

Supplement: Figure S1 — No obvious inhibitory effect in liver cancer cell proliferation upon treatment with extract of another Pleurotus mushroom, Pleurotus tuber-regium . MTT assay was applied with polysaccharide and protein complex from another mushroom Pleurotus tuber-regium (PTR) as control. (PDF) [file pone.0034406.s001.pdf]

**A**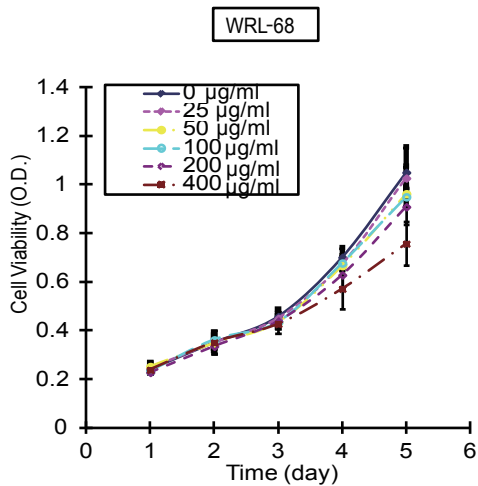**B**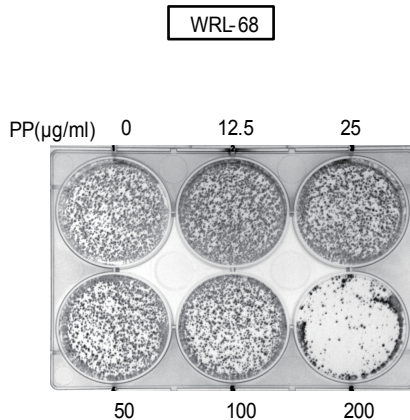

Supplement: Figure S2 — Potential cytotoxic activity of PP in normal cells. A. MTT assay was applied to determine the potential cytotoxic effect of PP against normal liver cell line WRL-68. B. Colony-formation assay demonstrated that WRL-68 cells are more resistant to PP than liver cancer cells. (PDF) [file pone.0034406.s002.pdf]

**A**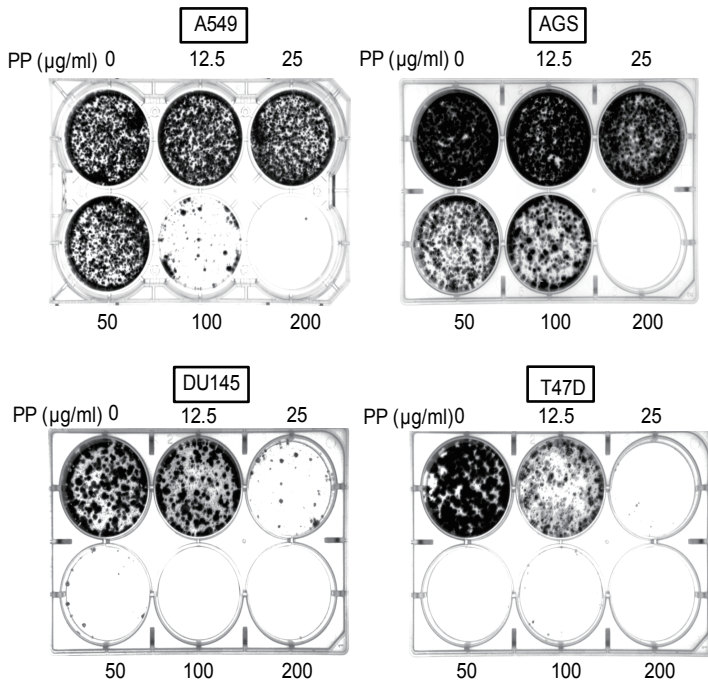

Supplement: Figure S3 — Effects of PP on the proliferation of other cancer cell lines. A. Colony-formation assay was used to test the anti-proliferative effect of PP in other cancer types, including lung cancer cells (A549), stomach cancer cells (AGS), prostate cancer cells (DU145) and breast cancer cells (T47D). (PDF) [file pone.0034406.s003.pdf]
